# Antiseptic Properties of Corrosive Sublimate in Minimal Quantities

**Published:** 1890-02

**Authors:** 


### Antiseptic Properties of Corrosive Sublimate in Minimal Quantities.

The powerful antiseptic properties of corrosive sublimate have only recently been fully appreciated, and experience has shown that equally positive results are obtainable with attenuated solutions as with dangerously strong ones.

Dr. Francois Scalji has recently been experimenting with very weak solutions of the sublimate and has found that when used at a temperature 115° to 125° F., solutions of a strength of 1:10,000, 1:20,000, and even 1:50,000, and 1:100,000, are capable of exerting germicidal actions equal to those of solutions of 1:1000 or 1:5000 at a lower temperature.

Dr. Scalji's experiments, which are reported in the *Bulletin Medicale*, October 2, 1889, were, briefly stated, as follows: First, he filled ten test tubes with fresh, normal urine. The first two of these were not treated; to the second pair a small quantity of a 1:1000 sublimate solution was added; to the third pair of tubes a similar quantity of a 1:5000 solution; to the fourth pair a 1:10,000, and to the last two, the same quantity of a 1:20,000 solution. Then one tube of each pair was kept at a temperature of from 59° to 77° F., and the others at a temperature from 115° to 125° F.

It was found that the two tubes to which no sublimate had been added soon began to undergo fermentation. The second pair, treated with the 1:1000 solution, remained completely sterile. With the third and fourth pairs similar results were obtained. Finally, the last two tubes were examined, and it was found that in the tube kept at a normal temperature fermentation had been retarded for a few days, whilst the tube kept at a temperature of 115° to 125° remained sterile after the lapse of a month. The experiment was frequently repeated and always yielded the same results.

Similar experiments were then made with meat juice and milk, and the results were identical with those obtained with urine.

Finally, the bacillus of erysipelas on culture of gelatine and blood serum were treated with weak solutions of sublimate, and it was observed that if the cultures were kept at a temperature of 150° or 125° sterilization was permanent and complete even when solutions of 1:50,000 or 1:100,000 had been employed; whereas, if the cultures were kept at a normal temperature, solutions of the above strength merely retarded their development for a few days.

The clinical value of these experiments is very evident, for, if satisfactory antiseptic results are obtainable with a 1:100,000 solution of corrosive sublimate, neither surgeon nor obstetrician need be circumspect regarding its free use. The irritant or caustic action of the stronger solutions will be absent, and the danger of poisoning through absorption entirely removed.

VALUE OF VACCINATION.—One of the most striking indications of the value of vaccination which has ever been given by a layman, is that which reached this country in the "Stanley Letters" of Nov. 25. Stanley writes: "The small-pox broke out among the Mayena and their followers, and the mortality was terrible. Our Zanzibaris escaped this pest, however, *owing to the vaccination they had undergone* on board the Madura."

PITTSBURG and Cincinnati now boil and filter their drinking water. It would be well for the dwellers in cities everywhere to follow their example.

